# Oesophageal Pain and Catecholamine Mediated Ecg Changes Mimicking Myocardial Ischaemia

**Published:** 1987-05

**Authors:** Robert F. Logan, Jim Shahi, John E. Sanderson

**Affiliations:** Department of Cardiology, Taunton and Somerset Hospital (Musgrove Park), Taunton, Somerset TA1 5DA; Department of Cardiology, Taunton and Somerset Hospital (Musgrove Park), Taunton, Somerset TA1 5DA; Department of Cardiology, Taunton and Somerset Hospital (Musgrove Park), Taunton, Somerset TA1 5DA

## Abstract

It is known that oesophageal pain can imitate angina and also that non specific ECG changes, probably catecholamine mediated, can be similar to those due to true myocardial ischaemia. Both of these can therefore pose a problem for the diagnosis of angina pain due to cardiac ischaemia. We report a patient who had both of these conditions simultaneously, pain on exertion appearing as angina but due to oesophagitis, and "ischaemic" ECG changes due to catecholamines—a double mimic of myocardial ischaemia.


### Oesophageal pain and catecholamine mediated ecg changes mimicking myocardial ischaemia

**Robert F. Logan, M.R.C.P.**

**Jim Shahi, M.R.C.P.**

**John E. Sanderson, M.D., M.R.C.P.**

Department of Cardiology, Taunton and Somerset Hospital (Musgrove Park), Taunton, Somerset TA1 5DA.

**ABSTRACT**

It is known that oesophageal pain can imitate angina and also that non specific ECG changes, probably catecholamine mediated, can be similar to those due to true myocardial ischaemia. Both of these can therefore pose a problem for the diagnosis of angina pain due to cardiac ischaemia. We report a patient who had both of these conditions simultaneously, pain on exertion appearing as angina but due to oesophagitis, and "ischaemic" ECG changes due to catecholamines—a double mimic of myocardial ischaemia.

**KEYWORDS**

Oesophageal pain, angina, ECG changes.

**INTRODUCTION**

Amongst the various pitfalls in the diagnosis of chest pain are mimicry of angina by oesophageal pain[1, 2] and the occurrence of catecholamine-mediated ECG changes resembling those due to ischaemia.[3] We report a patient who illustrated both of these problems simultaneously.

**CASE REPORT**

A 52 year old man was admitted with a six hour history of severe, "sharp" retrosternal chest pain, radiating to the arms, with sweating. It awoke him from sleep and was unresponsive to antacid and glyceryl trinitrate. Over the previous three weeks he had suffered attacks of similar pain on exercise, but also after meals and on reclining, in association with acid reflux. In 1979 he had suffered what was considered to be a small anterolateral subendocardial myocardial infarction.

On examination he was pain-free. The abnormal findings were a blood pressure of 180/110 mm Hg and grade one retinopathy. Initially his ECG was normal, but the following day biphasic T waves had developed across the anterior chest leads (Figure 1). These slowly resolved over six days. These ECG changes raised the possibility that the pain was cardiac and that he had had a myocardial infarction but cardiac enzymes were normal. Therefore further investigations were done. A barium swallow showed a sliding hiatus hernia with gastro-oesophageal reflux and irregularity of the lower oesophageal mucosa suggesting oesophagitis. A Bernstein acid perfusion test reproduced his pain which subsequently responded dramatically to Cimetidine and Gaviscon. However, a few days later his ECG abnormalities had returned. These were unaffected by beta blockade, but were corrected by infusion of either adrenaline (0.18mcg/Kg/min) or noradrenaline (0.18mcg/Kg/min). This effect was partially prevented when infusion was repeated during beta blockade. A treadmill exercise test was done using the modified Bruce protocol. The abnormal T wave changes which were present at rest became normal at Stage 1. At Stage 3 his typical chest pain developed but there was no ST segment depression and he continued to exercise until Stage 4. Thallium scanning at peak exercise and after rest was normal, with no reversible defects of perfusion.

Correspondence to: Dr. John Sanderson, Dept. of Cardiology, Taunton and Somerset Hospital (Musgrove Park), Taunton, Somerset TA1 5DA.

**COMMENT**

Although we did not do coronary angiography in this patient, it is unlikely that myocardial ischaemia was causing his pain because a negative exercise test and a normal thallium scan at peak exercise is considered to be a sensitive way of excluding significant coronary artery disease or myocardial ischaemia (4). Therefore we feel that his pain was oesophageal and it has been shown that a significant proportion of patients admitted with suspected ischaemic heart pain will have a purely oesophageal disorder (1, 5). The character, distribution, precipitating and relieving factors may all be "classically" cardiac. For example, as in our patient, oesophageal pain may be exercise-related, presumably due to mechanical induction of gastro-oesophageal reflux (2). No single test to prove an oesophageal cause for chest pain is entirely sensitive, and so extensive oesophageal investigation may be necessary (5). This may also be the case in patients with proven ischaemic heart disease whose pain responds poorly to treatment, since oesophageal disease may be co-existent, both conditions being common.

The diagnosis in our patient was further complicated by the abnormal ECG but the close similarity between catecholamine mediated and ischaemic repolarisation changes is well known. The catecholamine effect is not well understood. It may be a direct effect of raised catecholamine levels, or perhaps altered myocardial responsiveness. Three groups of patients have been described according to the ECG distribution of ST depression and T wave inversion, each group with a characteristic pattern of response to changes in catecholamine activity (3). In this patient the ECG changes were reversed completely by exercise and by an infusion of noradrenaline or adrenaline, indicating that they were likely to be due to a catecholamine imbalance rather than myocardial ischaemia. Thus, in the absence of significant ischaemia, this patient illustrates how closely oesophageal pain may mimic ischaemic heart pain, even being related to exercise, and how the diagnosis may be further confused by ischaemic-looking ECG changes which are in fact catecholamine mediated.

*(continued on page 50)*

### Abdominal symptoms in General Practice (Continued from page 34)

### REFERENCES

### Oesophageal pain and catecholamine mediated ecg changes (Continued from page 41)

### REFERENCES

1. BENNETT, J. R. (1983) Chest pain: heart or gullet? British Medical Journal, **286**, p.1231.
2. THORPE, J. A. C. (1983) Chest pain: heart or gullet? British Medical Journal, **286**, p.1899.
3. TAGGART, P., CARRUTHERS, M., JOSEPH, S., KELLY, H. B., MARCOMICHELAKIS, J., NOBLE, D., O'NEILL, G. and SOMERVILLE, W. (1979) Electrocardiographic changes resembling myocardial ischaemia in asymptomatic men with normal coronary arteriograms. British Heart Journal, **41**, p.214.
4. ILSLEY, C., STOCKLEY, A., CLITSAKIS, D. and LAYTON, C. (1982) Normal coronary arteriogram. An avoidable test? British Heart Journal, **48**, p.580.
5. BLACKWELL, J. N. and CASTELL, D. O. (1984) Oesophageal chest pain: a point of view. Gut, **25**, p.1.

