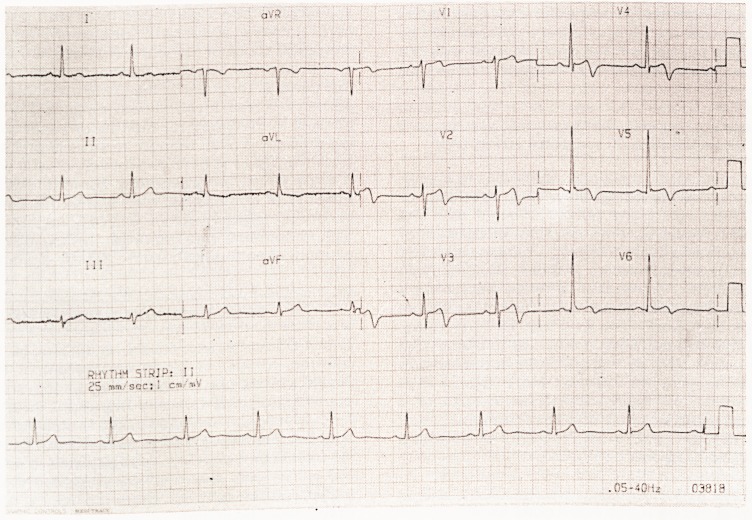# Oesophageal Pain Mimicking Cardiac Ischaemia

**Published:** 1987-08

**Authors:** 


					y^endum and apology
|l. "uuffi anu npuiugy
's ECG was unfortunately omitted from the article
Oesophageal Pain mimicking Cardiac ischaemia
by Logan, Shaw
Sanderson printed on p 41 of our last number Vol 102(ii).
??;'i' Trii 5 .*??] ?t Ii
25 -n^i'see;' ci i
a.-?
-?*fv_ifv_4^
' ,N | A i i ^ jj
j V6 i
0381
65

				

## Figures and Tables

**Figure f1:**